# Contribution of *SLC22A12* on hypouricemia and its clinical significance for screening purposes

**DOI:** 10.1038/s41598-019-50798-6

**Published:** 2019-10-07

**Authors:** Do Hyeon Cha, Heon Yung Gee, Raul Cachau, Jong Mun Choi, Daeui Park, Sun Ha Jee, Seungho Ryu, Kyeong Kyu Kim, Hong-Hee Won, Sophie Limou, Woojae Myung, Cheryl A. Winkler, Sung Kweon Cho

**Affiliations:** 1 Department of Pharmacology, Brain Korea 21 PLUS Project for Medical Sciences, Yonsei University College of Medicine, Seoul, Republic of Korea; 2 Advanced Biomedical Computational Science, Frederick National Laboratory for Cancer Research, National Cancer Institute, Frederick, MD USA; 3 Department of Laboratory Medicine, Green Cross, Yongin-si, Gyeonggi-do, Republic of Korea; 4 Department of Predictive Toxicology, Korea Institute of Toxicology, Daejeon, Republic of Korea; 5 Department of Epidemiology and Health Promotion and Institute for Health Promotion, Graduate School of Public Health, Yonsei University College of Medicine, Seoul, Republic of Korea; 6 Center for Cohort Studies, Total Healthcare Center, Kangbuk Samsung Hospital, Sungkyunkwan University School of Medicine, Seoul, Republic of Korea; 7 Department of Health Sciences and Technology, SAIHST, Sungkyunkwan University, Seoul, Republic of Korea; 8 Centre de Recherche en Transplantation et Immunologie (CRTI) UMR1064 Inserm, Université de Nantes, Nantes, France; 9 Institut de Transplantation en Urologie-Néphrologie (ITUN), Nantes University Hospital, Nantes, France; 10 Ecole Centrale de Nantes, Nantes, France; 11 Molecular Genetic Epidemiology Section, Basic Science Laboratory, Frederick National Laboratory for Cancer Research, Frederick, MD USA; 12 Department of Psychiatry, Seoul National University College of Medicine and Bundang Hospital, Seongnam, Korea; 13 Present Address: Molecular Genetic Epidemiology Section, Basic Research Laboratory, Center for Cancer Research, National Cancer Institute, 8560 Progress Drive, Frederick, MD 21701 USA

**Keywords:** Diagnostic markers, Predictive markers

## Abstract

Differentiating between inherited renal hypouricemia and transient hypouricemic status is challenging. Here, we aimed to describe the genetic background of hypouricemia patients using whole-exome sequencing (WES) and assess the feasibility for genetic diagnosis using two founder variants in primary screening. We selected all cases (N = 31) with extreme hypouricemia (<1.3 mg/dl) from a Korean urban cohort of 179,381 subjects without underlying conditions. WES and corresponding downstream analyses were performed for the discovery of rare causal variants for hypouricemia. Two known recessive variants within *SLC22A12* (p.Trp258*, pArg90His) were identified in 24 out of 31 subjects (77.4%). In an independent cohort, we identified 50 individuals with hypouricemia and genotyped the p.Trp258* and p.Arg90His variants; 47 of the 50 (94%) hypouricemia cases were explained by only two mutations. Four novel coding variants in *SLC22A12*, p.Asn136Lys, p.Thr225Lys, p.Arg284Gln, and p.Glu429Lys, were additionally identified. *In silico* studies predict these as pathogenic variants. This is the first study to show the value of genetic diagnostic screening for hypouricemia in the clinical setting. Screening of just two ethnic-specific variants (p.Trp258* and p.Arg90His) identified 87.7% (71/81) of Korean patients with monogenic hypouricemia. Early genetic identification of constitutive hypouricemia may prevent acute kidney injury by avoidance of dehydration and excessive exercise.

## Introduction

Uric acid (UA) is the final product of purine metabolism in humans^1^. After reuptake in the renal proximal tubule, only 10% of initially filtered UA is eliminated in the urine^2^. Serum UA level is determined by the balance between the rate of purine metabolism and clearance. Serum UA level converges to a normal distribution in general population^3^. At present, the heritability of serum urate has been estimated in several studies to account for 25% to 60% of the variance in serum UA level^4^. Common variants within *SLC2A9* and *ABCG2* were reported to be highly associated with serum UA levels with an additional 28 genetic loci affecting serum urate level in a genome-wide association study (GWAS) of more than 140,000 individuals of European ancestry^5^.

Hypouricemia, defined as extremely low serum UA level, is a rare condition which can be affected by malnutrition, and by genetic defects in critical pathways involving UA synthesis and reabsorption system. Deficiencies of xanthine dehydrogenase (XDH), Molybdenum Cofactor Sulfurase (MOCOS), purine nucleoside phosphorylase (PNP), and 5-phosphoribosyl-pyrophosphate (PRPP) are related to the defects in UA synthesis^6^. Renal hypouricemia (RHUC), with a prevalence of 0.19% to 0.53% in several studies, is diagnosed based on laboratory criteria as 1) hypouricemia (<2 mg/dL) and 2) increased fractional excretion of UA (>10%)^7^. RHUC is asymptomatic and rarely identified unless an individual presents with severe renal symptoms including exercise-induced acute kidney injury (EIAKI), renal failure and nephrolithiasis^8^. Despite these important clinical implications, differentiating between inherited and transient hypouricemia is challenging because a low level of UA may reflect malnutrition status, which can be resolved by genetic screening using a panel with well-established genetic variants^9^.

Two types of RHUC have been currently reported: type 1 (OMIM: 220150) caused by mutations in *SLC22A12* and type 2 (OMIM: 612076) caused by mutations in *SLC2A9*. A Japanese study first identified the protein-truncating p.Trp258* mutation in the *SLC22A12* gene, which encodes a drug transporter in the renal proximal tubule^10^. Recently, coding variants in *SLC22A12* and *SLC2A9* causal for RHUC has been reported in various ethnic groups including Israeli-Arab, Iraqi-Jewish, and Roma populations in the Czech Republic and Slovakia^7,11–15^.

In this study, we investigated unrelated subjects with extremely low levels of UA using whole-exome sequencing (WES) to identify monogenic coding variants responsible for RHUC, which could be used for genetic screening of RHUC in Asians. After the discovery of candidate variants, we performed direct genotyping of the most frequent mutations (p.Trp258* and p.Arg90His) in *SLC22A12* to replicate and quantify their contribution to RHUC in an independent Korean cohort, and to assess diagnostic feasibility of cost-effective genetic screening using these small subset of variants in hypouricemic patients.

## Results

### Hypouricemia prevalence and demographic information of 81 selected hypouricemic subjects

The prevalence of extreme hypouricemia (serum UA < 1.3 mg/dL) is 0.083% for the KoGES urban cohort (148/179,318). A total of 81 individuals (31 subjects in the KoGES urban cohort and 50 additional subjects from KCPS-II cohorts) were genetically tested for RHUC diagnostic assessment. Their baseline characteristics are summarized in Table 1. The 81 participants with RHUC (UA 0.74 ± 0.24 mg/dL; age, 47 ± 10 years; BMI, 23.3 ± 2.3 kg/m^2^; total cholesterol level, 189.2 ± 26.5 mg/dL) were healthy without chronic kidney disease, hypertension, diabetes mellitus or other metabolic diseases and without any history of smoking or malnutrition.

**Table 1:** Demographic characteristics.

Characteristics	Discovery group	Replication group	Total
	n = 31	n = 50	n = 81
Age (years)	47 ± 7	47 ± 12	47 ± 10
BMI^*^ (kg/m^2^)	23.5 ± 2.0	23.1 ± 2.5	23.3 ± 2.3
Waist circumstance, cm	79.1 ± 6.3	79.5 ± 9.3	79.2 ± 7.0
**Blood pressure**, **mmHg**			
Systolic	120 ± 13	117 ± 15	118 ± 14
Diastolic	74 ± 11	72 ± 11	73 ± 11
**Smoking status**			
Never smokers, no. (%)	31 (100.00)	33 (34.00)	64 (79.0)
Ever smokers, no. (%)	0 (0)	17 (66.00)	17 (21.0)
**Alcohol consumption**			
Never drinkers, no. (%)	19 (61.29)	18 (36.00)	37 (45.7)
Ever drinkers, no. (%)	12 (38.71)	32 (64.00)	44 (54.3)
Uric acid, mg/dL	0.77 ± 0.25	0.73 ± 0.24	0.74 ± 0.24
Total cholesterol, mg/dL	195.1 ± 25.4	185.5 ± 26.8	189.2 ± 26.5
Triglycerides, mg/dL	113.5 ± 66.6	113.8 ± 67.9	113.6 ± 67.0
Fasting glucose, mg/dL	90.2 ± 12.8	92.7 ± 21.2	91.7 ± 18.4
LDL cholesterol^¶^, mg/dL	115.5 ± 23.3	108.9 ± 22.0	111.4 ± 22.6
HDL cholesterol^†^, mg/dL	56.9 ± 12.3	55.0 ± 14.1	55.7 ± 13.4
Creatinine, mg/dL	0.78 ± 0.14	0.86 ± 0.17	0.83 ± 0.16

Values are presented as mean ± standard deviation (SD) for continuous data.

^*^The body mass index (BMI) was calculated as weight in kilograms divided by height in meters squared; ^¶^LDL Cholesterol: Low-Density Lipoprotein Cholesterol; HDL Cholesterol: ^†^High-Density Lipoprotein Cholesterol.

### Identification of coding variants in *SLC22A12* by whole-exome sequencing

**Figure 1:**
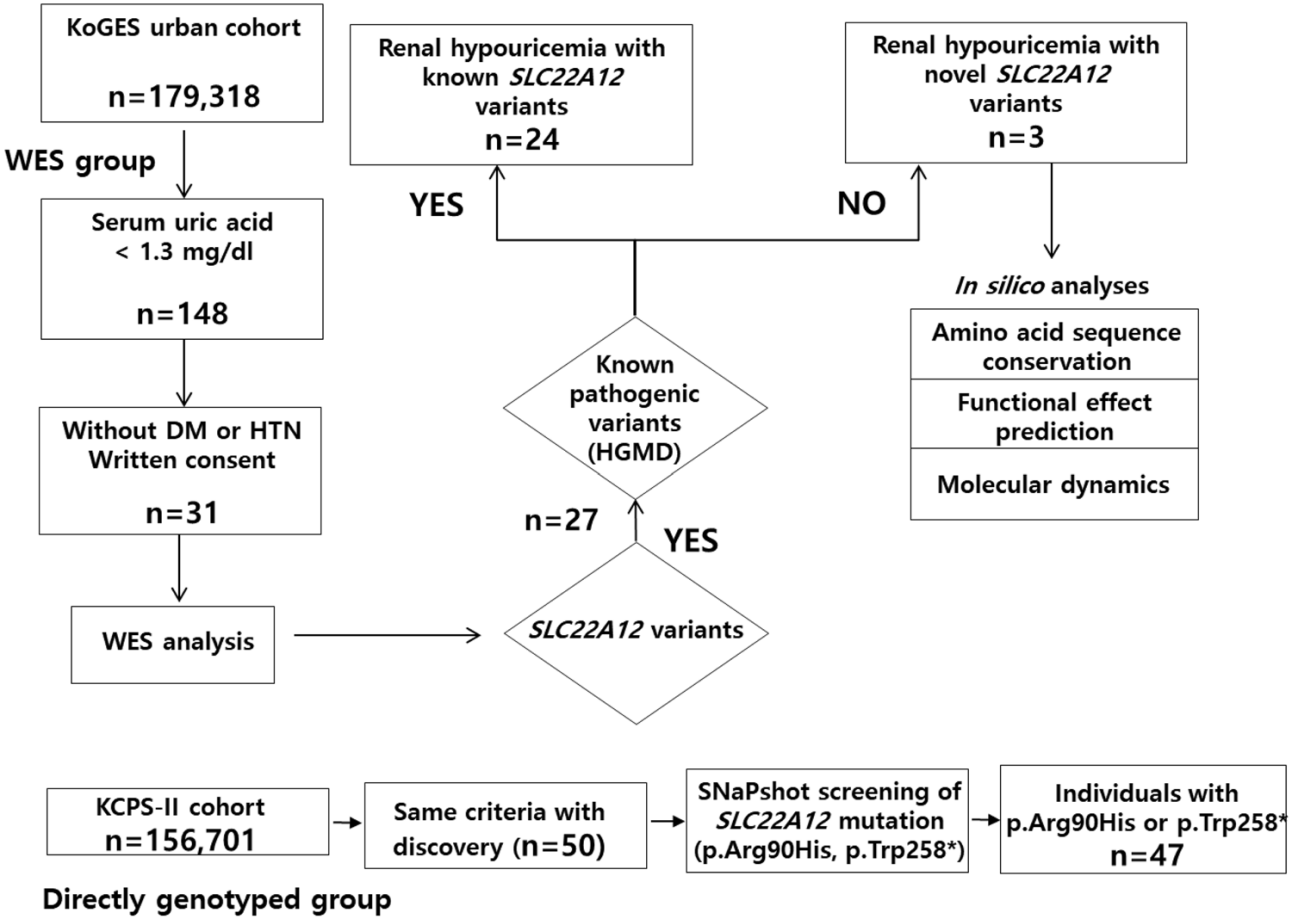
Overall flowchart for investigating novel variants associated with renal hypouricemia (DM: Diabetes Mellitus, HTN: Hypertension).

WES analysis was performed in 31 individuals with hypouricemia of the KoGES cohort (Fig. 1). The average depth coverage for these individuals was 85-fold. We performed variant calling and downstream filtering analyses assuming an autosomal recessive inheritance model. Coding variants in *SLC22A12* were observed in 87.1% (27/31) of the individuals (Table 2). One subject was a compound heterozygote for *SLC2A9* variants. In the remaining three individuals, variants within other genes were identified that appeared to have disease-causing potential and will be further investigated. 76% (24/31) individuals had variants previously reported in the Human Gene Mutation Database (HGMD) and the remaining 9.7% (3/31) exhibited novel missense mutations that had not been previously reported (Supplementary Table 3). 32.3% (10/31) of hypouricemia individuals were homozygous for the *SLC22A12* p.Trp258* resulting in a premature stop codon, the most reported disease-causing variant to date. We also identified two homozygous individuals for the *SLC22A12* p.Arg90His variants^16^. We found 12 compound heterozygous individuals for previously reported variants in the HGMD. In another individual, the p.Glu429Lys mutation was compound heterozygous with p.Trp258* in *SLC22A12* (NIH17A8865148). Two novel *SLC22A12* missense variants, p.Thr225Lys and p.Arg284Gln, were identified as compound heterozygotes (NIH17A8798528). Finally, we identified the novel p.Asn136Lys variant in the compound heterozygous state with the previously reported p.Leu418Arg variant (NIH17K4930892) (Supplementary Table 3).

**Table 2:** Distribution of *SLC22A12* variants in discovery and replication cohorts.

Number of Subjects (%)	Number of risk alleles in *SLC22A12*			Other than *SLC22A12*
	p.Trp258^*^	p.Arg90His	Others^*^	
**Discovery cohort**, **n = 31**				
10 (32.3%)	2	0	0	
7 (22.6%)	1	1	0	
2 (6.5%)	0	2	0	
5 (16.1%)	1	0	1	
3 (9.7%)	0	0	2	
	27 (87.1%)			4 (12.9%)
**Replication cohort**, **n = 50**				
10 (20.0%)	2	0	0	
22 (44.0%)	1	1	0	
1 (2.0%)	0	2	0	
10 (20.0%)	1	0	0	
4 (8.0%)	0	1	0	
	47 (94.0%)			3 (6.6%)

*p.Asn136Lys, p.Thr217Met, p.Gln382Leu, p.Leu418Arg, p.Glu429Lys or p.Arg477His.

Distribution of SLC22A12 variants in 27 hypouricemia individuals out of 31 in discovery cohort.

**Figure 2:**
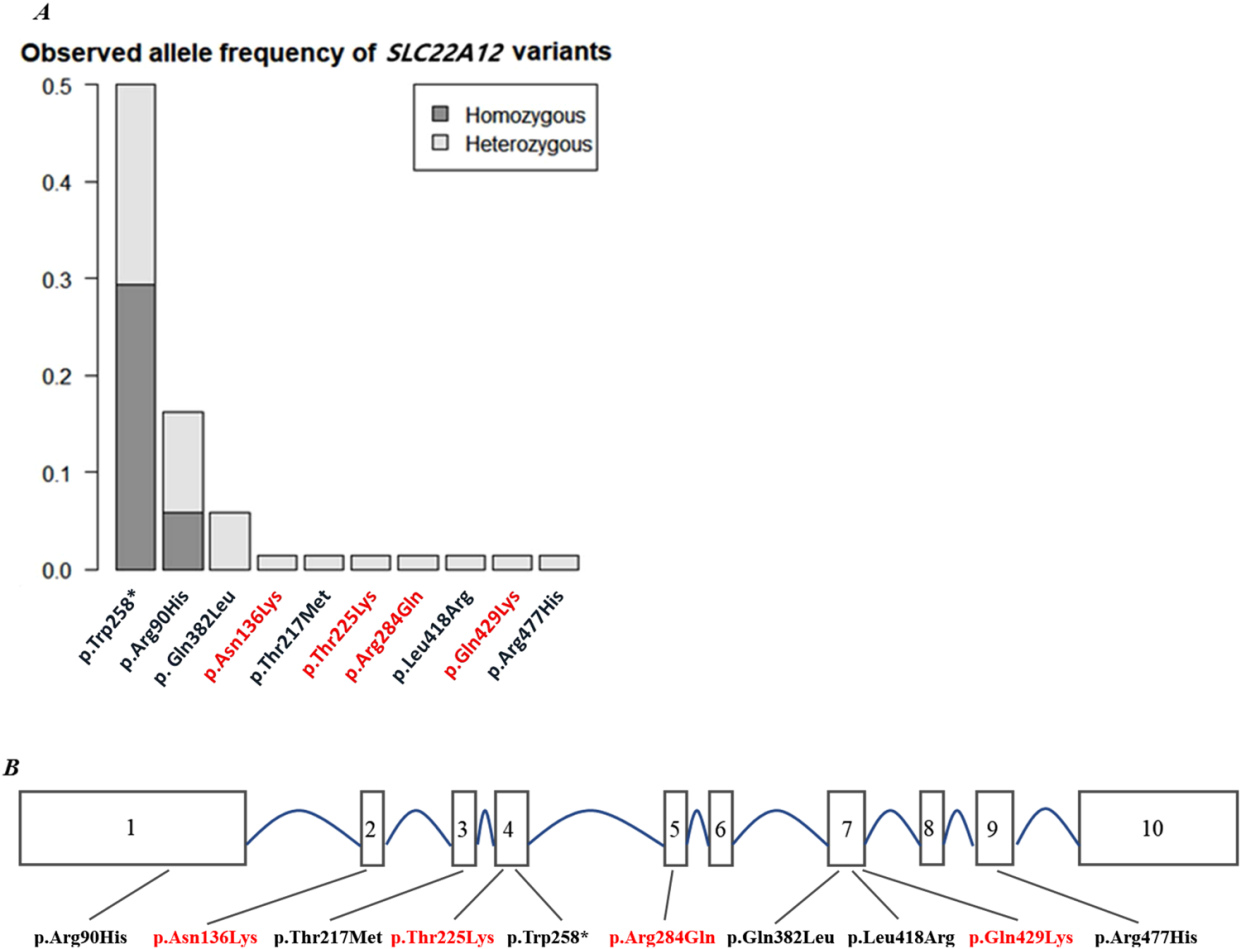
(**A**) Allele frequency distribution in SLC22A12 variants from KoGES cohorts (n = 31). (**B**) A schematic diagram of the exonic location of SLC22A12 variants found in 27 subjects. Newly discovered coding variants are marked in red.

The overall distribution of allele frequencies of the *SLC22A12* variants within our study is shown in Fig. 2. The novel *SLC22A12* variants were confirmed in the participant DNA samples by direct Sanger sequencing (Supplementary Fig. 1). Detailed properties of the four novel mutations in *SLC22A12* are shown in Table 3 and Supplementary Table 3. This information was collected by querying several methods for functional prediction (Mutation Taster, Polyphen-2, SIFT, Condel). All four tools predicted the two *SLC22A12* variants (p.Thr225Lys and p.Arg284Gln) reported in the NIH17A8798528 individual as deleterious. Amino acid sequence conservation was compared with *R*. *macaque*, *M*. *musculus*, *C*. *lupus familiaris*, and *L*. *africana* (Table 3). *SLC22A12* p.Glu429Lys is not conserved in *M*. *musculus* and p.Asn136Lysd is not conserved in *M*. *musculus*, *C*. *lupus familiaris*, and *L*. *africana*.

**Table 3:** Novel missense variants of *SLC22A12* identified in individuals with renal hypouricemia *via* whole-exome sequencing.

Gene symbol	Individual	Chr	Base position	Nucleotide change^a^	Amino acid change	Amino acid conservation				Frequency in the dbSNP database^b^	Frequency in the gnomAD database^c^	Mutation Taster^e^	PP2 Humvar^f^	SIFT^g^	Condel^h^
						*Rhesus* *macaque*	*Mus* *musculus*	*Canis lupus* *familiaris*	*Loxodonta* *africana*						
*SLC22A12*	NIH17A8865148	11	64367362	c.1285G > A	p.Glu429Lys	Glu	Gly	Glu	Glu	rs139140123 0.00005/5 (ExAC) 0.00008/1 (GO-ESP)	0.000044 (no homozygote)	DC	Bn (0.37)	Tol (0.05)	Neu (0.463)
	NIH17A8798528	11	64361119	c.674C > A	p.Thr225Lys	Thr	Thr	Thr	Thr	No	No	DC	Dam (0.998)	Del (0)	Del (0.919)
		11	64366008	c.851G > A	p.Arg284Gln	Arg	Arg	Arg	Arg	No	0.000019 (no homozygote)	DC	Dam (0.527)	Del (0.03)	Del (0.542)
	NIH17K4930892	11	64360256	c.408C > A	p.Asn136Lys	Asn	Asp	Asp	Asp	No	0.000004 (no homozygote)	PM	Bn (0.345)	Del (0)	Del (0.553)

Abbreviations are as follows: Chr, chromosome; Bn, benign; Condel, consensus deleteriousness score of non-synonymous single nucleotide variants; Dam, damaging; DC, disease causing; Del, deleterious; Neu, neutral; PM, polymorphism; PP2, PolyPhen-2 prediction score Humvar; SIFT, sorting intolerant from tolerant; SNP, single nucleotide polymorphism; Tol, tolerant. ^**a**^cDNA mutations are numbered according to human cDNA reference sequence NM_144585.2 (*SLC22A12*). ^b^dbSNP database (http://www.ncbi.nlm.nih.gov/SNP). ^c^gnomAD browser (http://gnomad.broadinstitute.org/). ^e^Mutation taster (http://www.mutationtaster.org/). ^f^PolyPhen-2 prediction score HumVar ranges from 0 to 1.0; 0 = benign, 1.0 = probably damaging (http://genetics.bwh.harvard.edu/pph2/). ^g^SIFT (http://sift.jcvi.org/). ^h^Condel (http://bbglab.irbbarcelona.org/fannsdb/).

### Molecular dynamic prediction of *SLC22A12* and novel variant location

**Figure 3:**
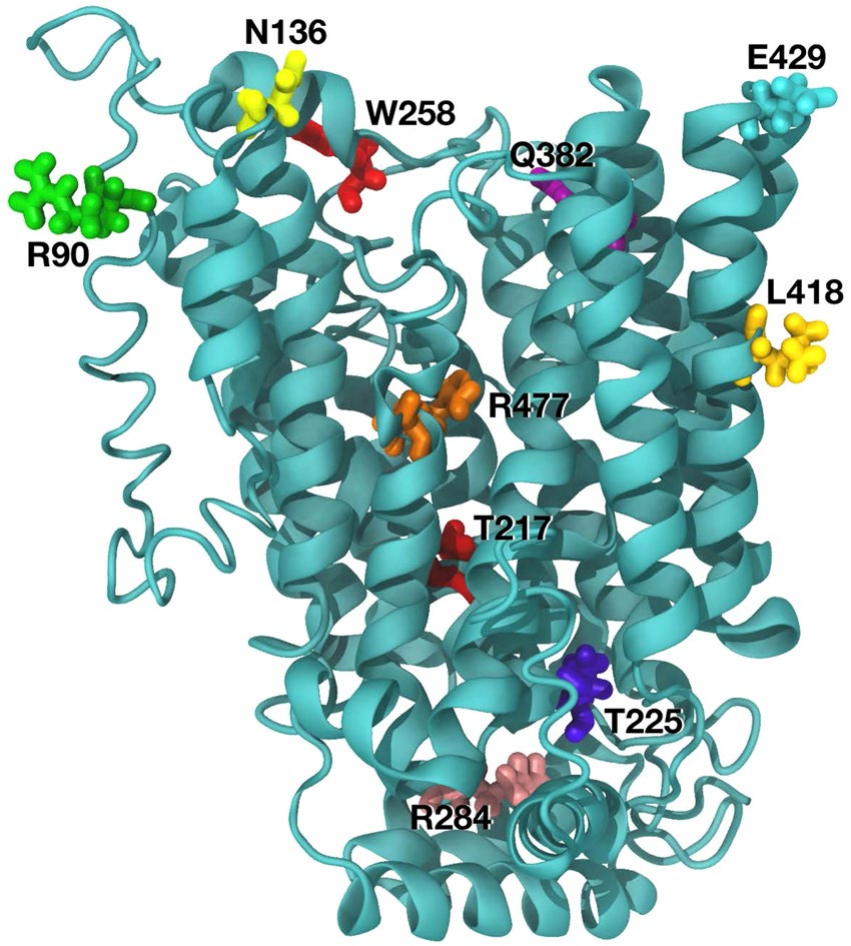
Residue mapping in the SLC22A12 predicted models.

The amino acid substitutions in *SLC22A12* (10 variants) were considered for a molecular dynamic prediction analysis. The predicted functional impact of the amino acid change is illustrated in Supplementary Table 4. Our overall organization of the *SLC22A12* protein was similar to the molecular dynamics approach described by Clemencon *et al*.^17^. Steered dynamic simulations of urate transport were performed with mutations in *SLC22A12* and are presented in Fig. 3. Assessing the extent of the effect of the variants in the S set is difficult in a qualitative analysis due to the large changes observed during the molecular dynamics trajectory. p.Arg90His, p.Thr217Met, p.Thr225Lys, p.Trp258*, and p.Leu418Arg for *SLC22A12* were predicted to alter protein structure defect. p.Arg284Gln and p.Arg477His were predicted to affect transport of uric acid. p.Asn136Lys and p.Gln382Leu for *SLC22A12* were predicted to affect binding of urate. *SLC22A12* p.Arg477His was predicted to both lower binding of urate and block the transportation pathway.

### Utility of screening with two genetic *SLC22A12* variants: c.774G > A (p.Trp258*) and c.269G > A (p.Arg90His)

Among 50 hypouricemia individuals from the KCPS-II replication cohort, 47 individuals carried at least one of these two genetic variants (Supplementary Table 2**)**: 10 individuals carried the c.774G > A (p.Trp258*) homozygous stop codon; one individual carried a c.269G > A (p.Arg90His) homozygous mutation; 22 individuals carried c.269G > A (p.Arg90His) and c.774G > A (p.Trp258*) in the compound heterozygous state; and 14 individuals carried either c.269G > A (p.Arg90His) or c.774G > A (p.Trp258*) heterozygous variants.

## Discussion

In this study, we comprehensively evaluated the contribution of *SLC22A12* to severe hypouricemia through WES of 31 RHUC cases and replication of two implicated SNVs in 50 RHUC cases for a total of 81 unrelated Korean subjects. This is the first study to evaluate causal genetic variants for their diagnostic potential for RHUC. Overall, our study confirmed the importance of two mutations (p.Trp258* and p.Arg90His) in *SLC22A12* for RHUC diagnosis found in 71/81(87.7%) of hypouricemia subjects.

Among the individuals exhibiting *SLC22A12* mutations, we described four novel variants that had not been previously reported in the HGMD: p.Asn136Lys, p.Thr225Lys, p.Arg284Gln, and p.Glu429Lys. p.Asn136Lys (exon2) was located at the end of an intracellular loop, p.Thr225Lys (exon4) was present at the beginning of an extracellular loop, p.Arg284Gln (exon5) was localized in the largest extracellular loop, and p.Glu429Lys, in which the distal end of exon 7 and the first part of exon 8 are connected *via* splicing, was found to be within the membrane before an intracellular loop (Fig. 2B.)^16^. p.Asn136Lys occurred together with p.Leu418Arg in the case of NIH17K4930892; however, we could not determine cis or trans configuration. p.Thr225Lys: p.Arg284Gln and p.Glu429Lys:p.Trp258* were found in the compound heterozygous state, respectively in in NIH17A8798528 and NIH17A8865148. None of these variants were not found in Japanese (OMIM #220150, RHUC type 1)^16,18–20^. Further studies are needed to elucidate the pathogenicity of rare variants of unknown significance located within novel genes in six unexplained cases. Family-based WES studies for cases not explained by the two founder variants in SLC22A12 might identify additional monogenic genes that cause extremely low serum UA levels.

Hypouricemia is often regarded as an unrecognized or neglected disorder from a public health aspect^21^. The prevalence of renal stone due to excess of UA excretion is 6–7 times higher in patients with RHUC than in individuals with normal uric acid levels^16^. Evidence of oxidative stress has accumulated not only in EIAKI and renal stone but also in neurodegenerative disease (e.g., Parkinson’s disease) in persons with RHUC, reflecting the ability of UA to act as a powerful scavenger of approximately 60% of peroxide radicals in the plasma^22–26^. The anti-oxidative stress hypothesis is also supported by the results of Facheris *et al*., which show that the *SLC2A9* mutation, associated with lower serum UA, increases the risk for early onset of neurodegenerative diseases^27^. Early identification and intervention of hypouricemia (avoidance of hard exercise, adequate hydration, and pre-emptively taking XO inhibitors) may prevent adverse events, especially among military personnel and athletics. XO inhibitor use (allopurinol or febuxostat) may be beneficial by lowering filtered UA. Screening of just two *SLC22A12* variants (p.Trp258*/rs121907892 and p.Arg90His/rs121907896) for soldiers or athletics will provide early diagnosis of inherited RHUC and increase awareness among primary care physicians and medical care professionals (e.g. military, sport physicians, urologists) of the potential adverse health outcomes in at-risk individuals.

Here, we have shown that two Asian founder variants can provide a precision molecular diagnosis for 90% of inherited hypouricemia in the homogeneous Korean population. Recently, large scale WES have identified novel variants in *SLC22A12* and *SLC2A9* in individuals with European ancestry^28^. Like other genetic traits and conditions, RHUC shows genetic allelic and locus heterogeneity. Given that genetic architecture and causal variants, particularly rare variants, differ among ethnic and racial groups, collaborative genomic research may identify novel, population-specific variants associated with RHUC. Considering all of the population-specific rare variants observed in hypouricemia patients in Japanese, Roma, and African populations, a cosmopolitan screening panel may yield high diagnostic power even among heterogeneous populations that present with complex genetic admixture.

In summary, this study indicates the cost-effectiveness of screening for just two variants to diagnosis monogenic renal hypouricemia, and its potential utility in at-risk groups.

## Materials and Methods

### Study participants

This study was approved by the institutional review board of the Kangbuk Samsung Hospital (IRB# KBSMC 2016-12-016). We screened the subjects in the Korean genome and epidemiology study (KoGES) – KoGES health examinee study (urban cohort) and KoGES twin and family study. Out of 179,318 individuals, we selected 31 (M:11, F:20) individuals of hypouricemia (<1.3 mg/dL) who exhibited no other syndromic features or secondary causes (chronic kidney disease, hypertension, diabetes mellitus or any other metabolic diseases) and without any history of smoking. We also excluded people who have poor nutrition status. We obtained genomic DNA samples from the National Biobank of Korea^29^. In addition, 50 additional hypouricemic subjects without secondary causes were selected from the Korean Cancer Prevention Study (KCPS-II) cohort from the Severance Hospital, Seoul, Korea (IRB#4-2011-0277)^30^. Whole-exome sequencing (WES) was done in first 31 individuals, whereas SNaPshot genotyping of two variants (p.Trp258* and p.Arg90His) within *SLC22A12* was performed to assess its screening purpose for second 50 subjects.

A total of 81 hypouricemic patients were therefore recruited for this study. All patients had given informed consent before they were enrolled in the study, which was conducted according to the Declaration of Helsinki. The overall flowchart for this study is presented in Fig. 1.

### DNA preparation and whole-exome sequencing

Genomic DNA was obtained from peripheral blood leukocytes. We checked the quality of the DNA with an OD260/280 ratio of 1.8–2.0 by 1% agarose gel electrophoresis and PicoGreen® dsDNA Assay (Invitrogen, Waltham, MA, USA). SureSelect sequencing libraries were prepared (Agilent SureSelect All Exon kit 50 Mb, Santa Clara, CA, USA) and the enriched library was then sequenced using the HiSeq 2500 sequencing system (Illumina, San Diego, CA, USA). Image analysis and base calling were performed with the pipeline software using default parameters. Mapping was done using the human reference genome assembly (GRCh37/hg19), and all variants were called and annotated using CLC Genomic Workbench (version 9.0.1) software (QIAGEN bioinformatics, Redwood city, CA, USA).

### WES variant filtering analysis

We performed variant-filtering analysis assuming an autosomal recessive or X-linked recessive pattern according to the predominantly observed inheritance mode in hereditary RHUC^31^. First, we systematically excluded variants with minor allele frequency (MAF) > 1%, which has been the conventional threshold for a rare variant, using dbSNP database (version 150), 1000 Genomes Projects phase 3 data (2,504 individuals), Exome Aggregation Consortium (ExAC, http://exac.broadinstitute.org), and Genome Aggregation Database (gnomAD, http://gnomad.broadinstitute.org/)^29^. Second, variants present in the homozygous or hemizygous state in in-house database consisting of 46 healthy Koreans without hypouricemia were excluded. Third, non-synonymous variants, small insertion/deletion (indel) or splice-site variants were selected. In the further analysis, we excluded single heterozygous variants so that only bi-allelic variants (homozygous, compound heterozygous, hemizygous for male) finally remained

### Direct Sanger sequencing

Confirmation of called variants was conducted *via* direct Sanger sequencing. The DNA sequences spanning the variants were amplified using specific primers (Supplementary Table 1) and sequenced using an Applied Biosystems 3500xl genetic analyzer3500XL (Applied Biosystems, Foster City, CA, USA).

### SNaPshot method

The SNaPshot assay of rs121907896 (p.Arg90His) and rs121907892 (p.Trp258*) was performed according to the manufacturer’s instructions (ABI PRISM SNaPshot Multiplex kit, Foster City, CA, USA). The analysis was carried out using GeneMapper software (version 4.0; Applied Biosystems). The primer sets for the SNaPshot assay are described in Supplementary Table 1.

### *In silico* analysis of novel missense variants

Prior to the analysis, known pathogenic variants of *SLC22A12* were screened in the Human Gene Mutation Database (HGMD^®^) as a public reference. For the newly discovered missense *SLC22A12* variants, we checked if the mutated amino acid resides are highly conserved across the vertebrate orthologs using the UCSC Genome Browser (https://genome.ucsc.edu/). Given the role of the nitrogen excretion function in the evolutionary process, we identified amino acid sequences in several mammals (*Rhesus macaque*, *Mus musculus*, *Canis lupus familiaris*, and *Loxodonta* a*fricana*) that share the urea cycle rather than direct UA excretion. Third, the prediction of the functional effect of missense variants was performed using the latest version of PolyPhen-2, SIFT, Condel, and Mutation Taster algorithms^32–35^.

### *In silico* prediction of molecular dynamics

We initially predicted the structure of *SLC22A12* using a homology modeling program, SWISS-MODEL (https://swissmodel.expasy.org/). The quality of predicted 3D structures was estimated on the basis of the geometrical analysis of the single model, global model quality estimation (GMQE) score and qualitative model energy analysis (QMEAN)^36^. The GenBank accession number used for each amino acid sequence was NP_653186 for *SLC22A12*. After homology modeling was completed, we selected a suitable SLC2A3 X-ray structure for *SLC22A12* (PDB ID: 4ZW9, *SLC2A3*)^37,38^. For the more stable molecular dynamics simulations, we used I-Tasser generated models^39^. All models were generated and made publicly available and can be recovered together with the statistics from the server site (https://zhanglab.ccmb.med.umich.edu/I-TASSER/about.html). All graphical representations were made using the initial I-Tasser generated models to aid reproducibility. A qualitative evaluation of the mutation effect was conducted based on four simple criteria. Binding urate (U) indicates the effect of the mutation on binding or urate because of the exposure of the mutated residue to the vestibular region or the urate binding motif cavity and/or involves a polar/nonpolar mutation affecting the interaction with urate. The structural effect (S) was evaluated as an increase in the root mean square displacement (RMSD) deviation computed during 25 ns of molecular dynamics (after 25 ns of equilibration) measured against the conformations obtained during a 25 ns trajectory for the initial sequence using either a solvated model or a Feedback Restrained Molecular Dynamics model (FRMD). FRMD affords a simple protocol to maximally retain structural features during a molecular dynamics trajectory while minimizing distortions imposed by an external restrain^40^. The transport effect (T) indicates that the mutation intrudes into the vestibular area blocking the possible passage of urate and is assigned based on a reduction of the internal cavity volume. We used all the models to identify geometries compatible with the mutation extending the initial molecular dynamic trajectory for *SLC22A12* (10 mutations) to 125 ns. All molecular dynamics calculations were performed using NAMD2^41^ and the ff99SB force field in the NVT ensemble with typical settings (T = 298 K, 2fs integration time, 12A cutoffs) obtained using QwikMD with default parameters to prepare the input files.

## Supplementary information

Supplementary tables and figures

## Data Availability

Before the official release, the data are available on reasonable request. The datasets generated during and/or analysed during the current study are available from the corresponding author on reasonable request. The data will be available at CODA (Clinical & Omics Data Archive, http://coda.nih.go.kr).
