# Supplementary material for: Contribution of SLC22A12 on hypouricemia and its clinical significance for screening purposes
Source: Sci Rep. 2019 Oct 7;9:14360. doi: 10.1038/s41598-019-50798-6 (PMC6779878; doi:10.1038/s41598-019-50798-6)

**Contribution of *SLC22A12* on hypouricemia and its clinical significance for screening purposes.**

Do Hyeon Cha^1^, Heon Yung Gee^1^, Raul Cachau^2^, Jong Mun Choi^3^, Daeui Park^4^, Sun Ha Jee^5^, Seungho Ryu^6^, Kyeong Kyu Kim^7^, Hong-Hee Won^7^, Sophie Limou^8,9,10,11^, Woojae Myung^12^, Cheryl A. Winkler^11^, and Sung Kweon Cho^7,13^

^1^Department of Pharmacology, Brain Korea 21 PLUS Project for Medical Sciences, Yonsei University College of Medicine, Seoul, Republic of Korea; ^2^Advanced Biomedical Computational Science, Frederick National Laboratory for Cancer Research, National Cancer Institute, Frederick, MD, USA; ^3^Department of Laboratory Medicine, Green Cross, Yongin-si, Gyeonggi-do, Republic of Korea; ^4^Department of Predictive Toxicology, Korea Institute of Toxicology, Daejeon, Republic of Korea; ^5^Department of Epidemiology and Health Promotion and Institute for Health Promotion, Graduate School of Public Health, Yonsei University College of Medicine, Seoul, Republic of Korea; ^6^Center for Cohort Studies, Total Healthcare Center, Kangbuk Samsung Hospital, Sungkyunkwan University School of Medicine, Seoul, Republic of Korea; ^7^Department of Health Sciences and Technology, SAIHST, Sungkyunkwan University, Seoul, Republic of Korea; ^8^Centre de Recherche en Transplantation et Immunologie (CRTI) UMR1064 Inserm, Université de Nantes, Nantes, France; ^9^Institut de Transplantation en Urologie-Néphrologie (ITUN), Nantes University Hospital, Nantes, France; ^10^Ecole Centrale de Nantes, Nantes, France; ^11^Basic Science Laboratory, Frederick National Laboratory for Cancer Research, Frederick, MD, USA; ^12^Department of Psychiatry, Seoul National University College of Medicine and Bundang Hospital, Seongnam, Korea; ^13^Basic Research Laboratory, Center for Cancer Research, National Cancer Institute, Frederick, MD, USA.

**Correspondence to**

**Sung Kweon Cho MD, PhD.**

Department of Health Sciences and Technology, SAIHST, Sungkyunkwan University,

Samsung Comprehensive Cancer Center (81, Irwon-ro, Gangnam-gu) Seoul, South Korea

(06351)

Tel: +82-2-2001-5137

Fax: +82-2-757-0436

E-mail: wontan@skku.edu

**Supplements**

Supplementary Table 1. Primer information for the *SLC22A12* variants

Supplementary Table 2. SNaPshot results of rs121907896 (p.Arg90His) and rs121907892 (p.Trp258*)

Supplementary Table 3. Bi-allelic mutations of *SLC22A12* in 27 individuals with hypouricemia

Supplementary Table 4. Predicted functional impact of amino acid change

Supplementary Table 5. Variant filtering process of whole-exome sequencing (WES)

Supplementary Figure 1. Sanger-sequencing traces of novel variants detected in *SLC22A*

**Supplementary Table 1. Primer information for the *SLC22A12* variants.**

| ***SLC22A12*** | p.Trp258* | Forward | TTGCAGTGATGGAGTGGAC |
| --- | --- | --- | --- |
|  |  | Reverse | CTCCTGGCTTTCCTCCAG |
|  | p.Arg90His | Forward | ATGGCTCTGATGGTCTCC |
|  |  | Reverse | AGATGCTGCGGTCATAGA |
|  | p.Asn136Lys | Forward | CAATAGGGAGCCATGGAAGA |
|  |  | Reverse | CTTGGTTTTGTGAGGCTGGT |
|  | p.Thr225Lys | Forward | TCAGATGGCTGTGATGGGTA |
|  |  | Reverse | AGGAAGAAGGGGACCGAGAC |
|  | p.Arg284Gln | Forward | GGAGCAGTGGGTACAGGGTA |
|  |  | Reverse | AGGACCCAGCCTTACCTCAG |
|  | p.Glu429Lys | Forward | TGGACATCCCAGCCAAGAT |
|  |  | Reverse | CTAGTCCAGCACCTCCAAGG |

**Supplementary Table 2. SNaPshot results of *SLC22A12* rs121907896 (p.Arg90His) and rs121907892 (p.Trp258*) in the replication cohort**

| **Subject ID** | **rs121907896**  **(p.Arg90His ; G>A)** | **rs121907892**  **(p.Trp258*; G>A)** |
| --- | --- | --- |
| 1103762 | GG | GA |
| 1802477 | GG | GA |
| 1303740 | GG | GA |
| 1101727 | GG | GA |
| 1108995 | GG | GA |
| 1203374 | GA | GG |
| IB03748 | GA | GG |
| IB09509 | GG | GA |
| IB00225 | GA | GG |
| B504463 | GA | GG |
| 193617 | GG | GA |
| NIH17K4324007 | GG | GA |
| NIH17K4697793 | GG | GA |
| NIH17K4577782 | GG | GA |
| 1110890 | GA | GA |
| IB06273 | GA | GA |
| 1107408 | GA | GA |
| 1107096 | GA | GA |
| 1304052 | GA | GA |
| 1302122 | GA | GA |
| 1101730 | GA | GA |
| 1800904 | GA | GA |
| 1306604 | GA | GA |
| IB05053 | GA | GA |
| IB02052 | GA | GA |
| S501819 | GA | GA |
| S501875 | GA | GA |
| S511522 | GA | GA |
| S406644 | GA | GA |
| 163064 | GA | GA |
| 799334 | GA | GA |
| NIH17K4518600 | GA | GA |
| NIH17K4132316 | GA | GA |
| NIH17K4943218 | GA | GA |
| NIH17K4691132 | GA | GA |
| NIH17K4358876 | GA | GA |
| 9900585 | GG | GG |
| 632847 | GG | GG |
| NIH17K4930892 | GG | GG |
| 1501793 | GG | AA |
| 1200777 | GG | AA |
| 1803940 | GG | AA |
| 1107800 | GG | AA |
| B500348 | GG | AA |
| B503093 | GG | AA |
| S601007 | GG | AA |
| S408073 | GG | AA |
| 14689 | GG | AA |
| NIH17K4365303 | GG | AA |
| 713416 | AA | GG |

**Supplementary Table 3. Bi-allelic mutations of *SLC22A12* in 27 individuals from the discovery cohort**

| **Individual** | **Gene** | **Zygosity** | **Genomic**  **change** | **Amino acid**  **change** | **Location** | **Mutation**  **type** | **HGMD®** |
| --- | --- | --- | --- | --- | --- | --- | --- |
| NIH17A8087761 | *SLC22A12* | Homozygous | c.774G>A | p.Trp258* | Exon 4 | Loss-of-function | Yes |
| NIH17A8113116 | *SLC22A12* | Homozygous | c.774G>A | p.Trp258* | Exon 4 | Loss-of-function | Yes |
| NIH17A8172895 | *SLC22A12* | Homozygous | c.774G>A | p.Trp258* | Exon 4 | Loss-of-function | Yes |
| NIH17A8208690 | *SLC22A12* | Homozygous | c.774G>A | p.Trp258* | Exon 4 | Loss-of-function | Yes |
| NIH17A8214059 | *SLC22A12* | Homozygous | c.774G>A | p.Trp258* | Exon 4 | Loss-of-function | Yes |
| NIH17A8441978 | *SLC22A12* | Homozygous | c.774G>A | p.Trp258* | Exon 4 | Loss-of-function | Yes |
| NIH17A8503979 | *SLC22A12* | Homozygous | c.774G>A | p.Trp258* | Exon 4 | Loss-of-function | Yes |
| NIH17A8555604 | *SLC22A12* | Homozygous | c.774G>A | p.Trp258* | Exon 4 | Loss-of-function | Yes |
| NIH17A8900052 | *SLC22A12* | Homozygous | c.774G>A | p.Trp258* | Exon 4 | Loss-of-function | Yes |
| NIH17A8951027 | *SLC22A12* | Homozygous | c.774G>A | p.Trp258* | Exon 4 | Loss-of-function | Yes |
| NIH17A8293564 | *SLC22A12* | Homozygous | c.269G>A | p.Arg90His | Exon 1 | Missense | Yes |
| NIH17A8668884 | *SLC22A12* | Homozygous | c.269G>A | p.Arg90His | Exon 1 | Missense | Yes |
| NIH17A8265785 | *SLC22A12* | Compound  heterozygous | c.774G>A  c.269G>A | p.Trp258*  p.Arg90His | Exon 4  Exon 1 | Loss-of-function  Missense | Yes  Yes |
| NIH17A8304859 | *SLC22A12* | Compound  heterozygous | c.774G>A  c.269G>A | p.Trp258*  p.Arg90His | Exon 4  Exon 1 | Loss-of-function  Missense | Yes  Yes |
| NIH17A8429119 | *SLC22A12* | Compound  heterozygous | c.774G>A  c.269G>A | p.Trp258*  p.Arg90His | Exon 4  Exon 1 | Loss-of-function  Missense | Yes  Yes |
| NIH17A8517296 | *SLC22A12* | Compound  heterozygous | c.774G>A  c.269G>A | p.Trp258*  p.Arg90His | Exon 4  Exon 1 | Loss-of-function  Missense | Yes  Yes |
| NIH17A8595401 | *SLC22A12* | Compound  heterozygous | c.774G>A  c.269G>A | p.Trp258*  p.Arg90His | Exon 4  Exon 1 | Loss-of-function  Missense | Yes  Yes |
| NIH17A8768605 | *SLC22A12* | Compound  heterozygous | c.774G>A  c.269G>A | p.Trp258*  p.Arg90His | Exon 4  Exon 1 | Loss-of-function  Missense | Yes  Yes |
| NIH17A8775970 | *SLC22A12* | Compound  heterozygous | c.774G>A  c.269G>A | p.Trp258*  p.Arg90His | Exon 4  Exon 1 | Loss-of-function  Missense | Yes  Yes |
| NIH17A8850018 | *SLC22A12* | Compound  heterozygous | c.774G>A  c.650C>T | p.Trp258*  p.Thr217Met | Exon 4  Exon 3 | Loss-of-function  Missense | Yes  Yes |
| NIH17A8449886 | *SLC22A12* | Compound  heterozygous | c.774G>A  c.1145A>T | p.Trp258*  p.Gln382Leu | Exon 4  Exon 7 | Loss-of-function  Missense | Yes  Yes |
| NIH17A8615650 | *SLC22A12* | Compound  heterozygous | c.774G>A  c.1145A>T | p.Trp258*  p.Gln382Leu | Exon 4  Exon 7 | Loss-of-function  Missense | Yes  Yes |
| NIH17A8656939 | *SLC22A12* | Compound  heterozygous | c.774G>A  c.1145A>T | p.Trp258*  p.Gln382Leu | Exon 4  Exon 7 | Loss-of-function  Missense | Yes  Yes |
| NIH17A8304077 | *SLC22A12* | Compound  heterozygous | c.1145A>T  c.1430G>A | p.Gln382Leu  p.Arg477His | Exon 7  Exon 9 | Missense  Missense | Yes  Yes |
| NIH17A8865148 | *SLC22A12* | Compound  heterozygous | c.774G>A  c.1285G>A | p.Trp258*  p.Glu429Lys | Exon 4  Exon 7-8 | Loss-of-function  Missense | Yes  **No** |
| NIH17A8798528 | *SLC22A12* | Compound  heterozygous | c.674C>A  c.851G>A | p.Thr225Lys  p.Arg284Gln | Exon 4  Exon 5 | Missense  Missense | **No**  **No** |
| NIH17K4930892 | *SLC22A12* | Compound  heterozygous | c.408C>A  c.1253T>G | p.Asn136Lys  p.Leu418Arg | Exon 2  Exon 7 | Missense  Missense | **No**  Yes |

HGMD®: The Human Gene Mutation Database

**Supplementary Table 4. Predicted functional impact of amino acid change**

| Gene | Chromosome | Base change | Amino acid  change | Assigned effect | FRMD | RMSD |
| --- | --- | --- | --- | --- | --- | --- |
| *SLC22A12* | 11 | 269G>A | p.Arg90His R90H | S | 2.3 | >4.5 |
|  | 11 | 408C>A | p.Asn136Lys N136K | B | 2.0 | 3.2 |
|  | 11 | 650C>T | p.Thr217Met T217M | S | 3.1 | >4.7 |
|  | 11 | 674C>A | p.Thr225Lys T225K | S | 2.5 | >4.0 |
|  | 11 | 774G>A | p.Trp258* W258X | S |  | >3.9 |
|  | 11 | 851G>A | p.Arg284Gln R284Q | T | 1.8 | 2.2 |
|  | 11 | 1145A>T | p.Gln382Leu Q382L | B | 2.0 | 3.0 |
|  | 11 | 1253T>G | p.Leu418Arg L418R | S | 4.3 | >5.3 |
|  | 11 | 1285G>A | p.Glu429Lys E429K | S | 6.1 | >5.5 |
|  | 11 | 1430G>A | p.Arg477His R477H | T | 1.5 | 2.0 |

U, Binding urate; S, Structural effect; T, Transport effect ; FRMD : Feedback Restrained Molecular Dynamics model ; RMSD : the root-mean-square deviation of atomic positions

| **Supplementary Table 5. Variant filtering process of whole-exome sequencing (WES).** | | | | | | | | | | |
| --- | --- | --- | --- | --- | --- | --- | --- | --- | --- | --- |
| **Individual** | **Total sequence reads** | **Matched reads** | **Total number of variants detected** | **Variants with**  **MAF <1% (dbSNP)** | **Variants**  **filtered by**  **46 controls** | **Non-synonymous**  **and**  **splice site variants** | **Located within**  **splice sites** | **Stop codon**  **gained or lost** | **Insertion**  **or**  **Deletion** | **Missense** |
| NIH17A8004492 | 64,404,038 | 63,765,823 (99.0%) | 152,116 | 30,685 | 18,940 | 1,412 | 102 | 22 | 113 | 1,175 |
| NIH17A8087761 | 56,676,376 | 56,111,878 (99.0%) | 146,634 | 29,051 | 18,187 | 1,372 | 80 | 29 | 106 | 1,157 |
| NIH17A8113116 | 68,701,420 | 67,982,670 (99.0%) | 158,718 | 32,581 | 20,146 | 1,406 | 94 | 19 | 109 | 1,184 |
| NIH17A8172895 | 73,113,446 | 71,606,666 (97.9%) | 162,283 | 34,440 | 21,724 | 1,479 | 104 | 24 | 135 | 1,216 |
| NIH17A8208690 | 64,670,324 | 63,992,902 (99.0%) | 153,913 | 31,307 | 19,568 | 1,364 | 106 | 20 | 105 | 1,133 |
| NIH17A8214059 | 58,124,176 | 57,495,669 (98.9%) | 145,535 | 29,824 | 18,534 | 1,395 | 77 | 30 | 112 | 1,176 |
| NIH17A8239849 | 68,750,852 | 68,019,481 (98.9%) | 156,924 | 32,110 | 19,964 | 1,455 | 104 | 25 | 126 | 1,200 |
| NIH17A8265785 | 66,823,180 | 66,155,417 (99.0%) | 159,843 | 32,211 | 20,082 | 1,381 | 86 | 19 | 137 | 1,139 |
| NIH17A8293564 | 69,894,594 | 69,195,093 (99.0%) | 155,697 | 32,202 | 20,270 | 1,378 | 94 | 25 | 95 | 1,164 |
| NIH17A8304077 | 79,754,406 | 78,826,485 (98.8%) | 169,250 | 34,872 | 21,778 | 1,407 | 116 | 23 | 124 | 1,144 |
| NIH17A8304859 | 68,093,668 | 67,442,778 (99.0%) | 154,075 | 30,431 | 18,678 | 1,373 | 89 | 34 | 122 | 1,128 |
| NIH17A8429119 | 73,145,442 | 72,434,184 (99.0%) | 159,372 | 33,268 | 20,693 | 1,336 | 98 | 23 | 105 | 1,110 |
| NIH17A8441978 | 71,376,474 | 70,645,731 (99.0%) | 161,415 | 32,762 | 20,256 | 1,426 | 99 | 23 | 101 | 1,203 |
| NIH17A8449886 | 66,608,590 | 65,938,984 (99.0%) | 158,473 | 31,920 | 19,682 | 1,463 | 79 | 27 | 120 | 1,237 |
| NIH17A8503979 | 74,272,648 | 73,486,802 (98.9%) | 160,278 | 33,188 | 20,457 | 1,508 | 110 | 29 | 136 | 1,233 |
| NIH17A8517296 | 68,884,982 | 68,162,138 (99.0%) | 158,557 | 32,947 | 20,485 | 1,425 | 106 | 24 | 128 | 1,167 |
| NIH17A8555604 | 67,513,014 | 66,846,675 (99.0%) | 156,163 | 31,574 | 19,671 | 1,393 | 95 | 23 | 112 | 1,163 |
| NIH17A8568242 | 64,956,500 | 64,301,552 (99.0%) | 155,946 | 32,088 | 19,949 | 1,422 | 105 | 24 | 129 | 1,164 |
| NIH17A8595401 | 71,668,456 | 70,908,151 (98.9%) | 162,338 | 33,746 | 21,155 | 1,391 | 91 | 24 | 110 | 1,166 |
| NIH17A8615650 | 71,338,744 | 70,601,040 (99.0%) | 160,157 | 32,454 | 20,194 | 1,422 | 110 | 30 | 119 | 1,163 |
| NIH17A8656939 | 62,979,222 | 62,313,244 (98.9%) | 153,112 | 31,518 | 19,611 | 1,478 | 90 | 36 | 117 | 1,235 |
| NIH17A8668884 | 60,942,542 | 60,345,439 (99.0%) | 151,139 | 30,694 | 19,075 | 1,364 | 113 | 17 | 113 | 1,121 |
| NIH17A8738324 | 69,795,198 | 69,020,373 (98.9%) | 159,037 | 33,273 | 20,793 | 1,438 | 96 | 31 | 111 | 1,200 |
| NIH17A8768605 | 58,721,580 | 58,147,016 (99.0%) | 147,337 | 29,956 | 18,720 | 1,478 | 81 | 22 | 137 | 1,238 |
| NIH17A8775970 | 63,025,820 | 62,345,842 (98.9%) | 153,453 | 31,567 | 19,850 | 1,453 | 107 | 26 | 106 | 1,214 |
| NIH17A8798528 | 70,890,838 | 70,084,565 (98.9%) | 160,802 | 33,621 | 20,912 | 1,285 | 99 | 29 | 114 | 1,143 |
| NIH17A8850018 | 77,931,600 | 77,085,301 (98.9%) | 164,000 | 33,796 | 21,148 | 1,501 | 117 | 28 | 131 | 1,225 |
| NIH17A8865148 | 68,084,478 | 67,389,246 (99.0%) | 156,664 | 32,509 | 20,246 | 1,395 | 107 | 17 | 102 | 1,169 |
| NIH17A8900052 | 65,485,846 | 64,787,659 (98.9%) | 154,997 | 31,513 | 19,709 | 1,382 | 98 | 26 | 115 | 1,143 |
| NIH17A8951027 | 67,293,984 | 66,595,784 (99.0%) | 155,438 | 31,875 | 19,893 | 1,384 | 87 | 30 | 102 | 1,165 |
| NIH1705180563 | 68,013,252 | 67,232,710 (98.9%) | 157,342 | 32,234 | 20,184 | 1,555 | 146 | 17 | 122 | 1,270 |

**Supplementary Figure 1. Sanger-sequencing traces of variants detected in *SLC22A12***


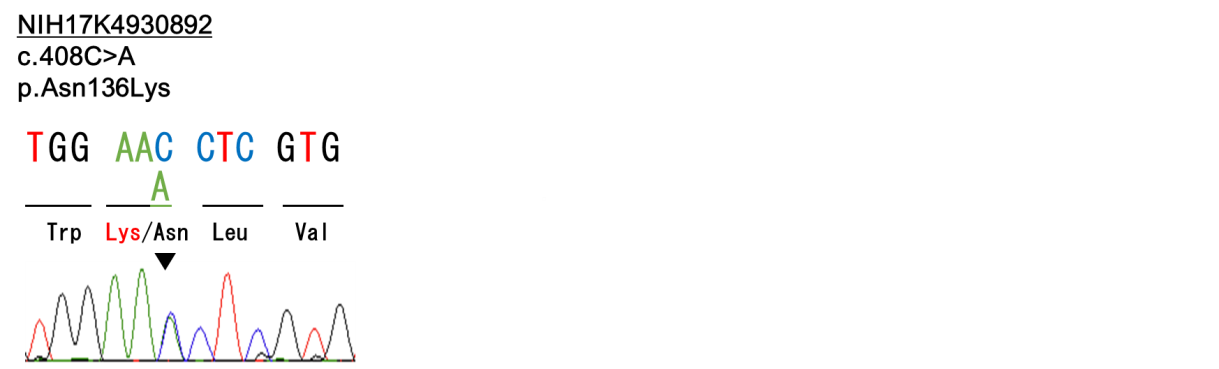

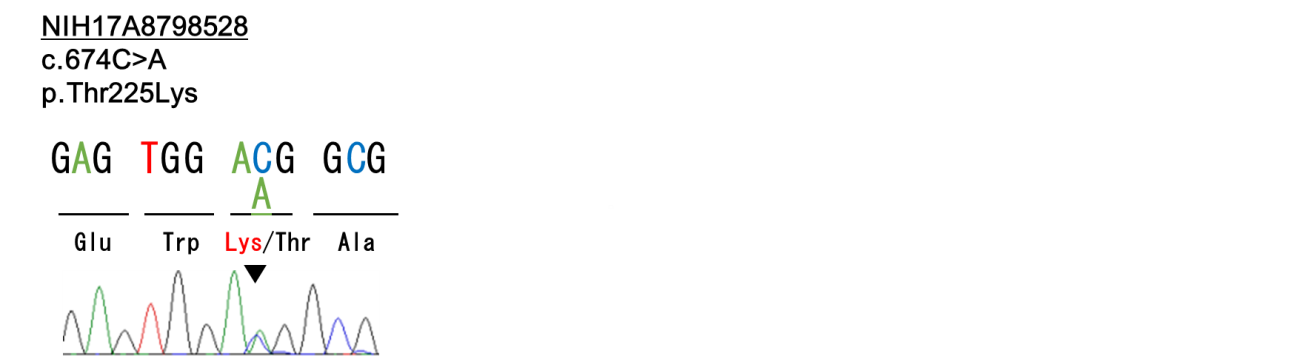

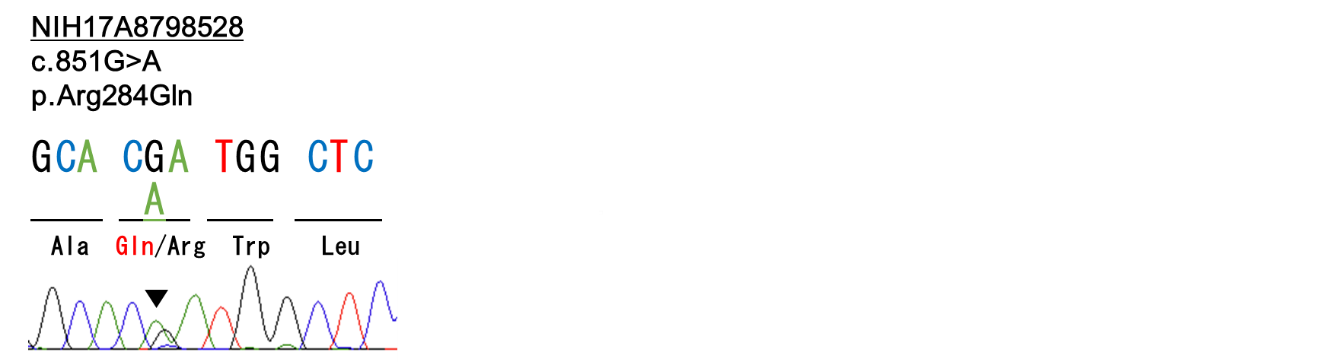

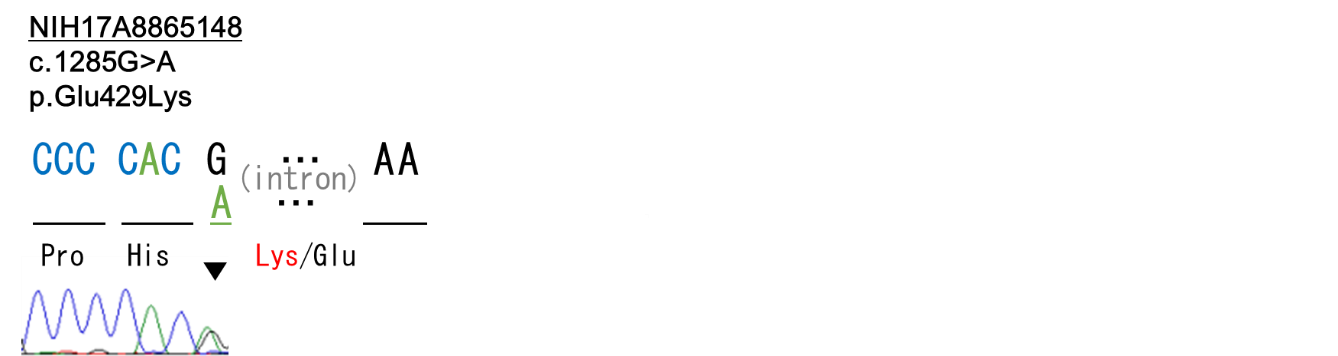

Supplement: Supplementary file 1 — Supplementary tables and figures [file 41598_2019_50798_MOESM1_ESM.docx]
